# The Sporobiota of the Human Gut

**DOI:** 10.1080/19490976.2020.1863134

**Published:** 2021-01-07

**Authors:** Muireann Egan, Eugene Dempsey, C. Anthony Ryan, R. Paul Ross, Catherine Stanton

**Affiliations:** a Food Biosciences Department, Teagasc Food Research Centre, Moorepark, Fermoy, Co. Cork, Ireland; b APC Microbiome Ireland, University College Cork, Cork, Ireland; c Department of Neonatology, Cork University Maternity Hospital, Cork, Ireland

**Keywords:** Gut microbiome, endospores, early life, transmission, sporobiota, clostridia, infant formula, breast milk

## Abstract

The human gut microbiome is a diverse and complex ecosystem that plays a critical role in health and disease. The composition of the gut microbiome has been well studied across all stages of life. In recent years, studies have investigated the production of endospores by specific members of the gut microbiome. An endospore is a tough, dormant structure formed by members of the Firmicutes phylum, which allows for greater resistance to otherwise inhospitable conditions. This innate resistance has consequences for human health and disease, as well as in biotechnology. In particular, the formation of endospores is strongly linked to antibiotic resistance and the spread of antibiotic resistance genes, also known as the resistome. The term sporobiota has been used to define the spore-forming cohort of a microbial community. In this review, we present an overview of the current knowledge of the sporobiota in the human gut. We discuss the development of the sporobiota in the infant gut and the perinatal factors that may have an effect on vertical transmission from mother to infant. Finally, we examine the sporobiota of critically important food sources for the developing infant, breast milk and powdered infant formula.

## Introduction

Bacteria face a number of challenges and stresses from their outside environment, including but not limited to, extremes in temperature, water and nutrient deprivation, oxygen, antibiotics and UV radiation. In response to these challenges, bacteria can alter their gene expression to produce proteins involved in oxidative or acid stress response, DNA repair and antibiotic resistance.^1^ Alternatively, a select cohort of bacteria have the ability to produce endospores, a highly stress-resistant but metabolically dormant state which allows for survival and spread under otherwise inhospitable conditions. Initiation of the sporulation process can occur under the conditions mentioned above, such as low pH, nutrient deprivation or exposure to oxygen.^2,3^ Germination, the return to vegetative growth, is induced by nutrients and other agents called germinants.^4^ Endospores are produced by members of the Firmicutes, a large, diverse and morphologically complex bacterial phylum.^5^ Within this phylum, the genus *Bacillus* has been used as a model organism for the study of endospore formation^3,4^ and spore-forming *Clostridioides difficile* represent a significant challenge due to their pathogenicity.^6^ However, it should be noted that endospore-formation is not limited to just the Bacilli and Clostridia classes.^7^ While the Firmicutes phylum is mostly comprised of Gram-positive bacteria, the Negativicutes class stains Gram-negative and yet shares a number of sporulation genes with *Clostridia* spore-formers.^8^ Indeed, studies of the sporulation process in *Acetonema longum*, a Gram-negative member of the Veillonellaceae family in the Firmicutes phylum, indicate that their outer membrane is formed by the inversion of the inner membrane during sporulation. The authors suggest that the Gram-negative outer membrane may have originated from the sporulation process.^9^ Table 1 outlines the phylogeny of bacteria from the Firmicutes phylum discussed throughout this review. However, endospores are not the only form of spore produced by bacteria. The genus *Streptomyces* of the Actinobacteria phylum produces exospores in response to nutrient limitation.^10^ During vegetative growth, *Streptomyces* grow as multicellular branching filamentous hyphae. The formation of the exospore in *Streptomyces* begins extracellularly, as non-branching aerial hyphae form from the colony surface.^10,11^ Myxospores are a third type of spore produced by *Myxococcus xanthus*, a Gram-negative bacterium from the Proteobacteria phylum. In *M. xanthus*, spores are formed by the rearrangement of the rod-shaped vegetative cell to a spherical spore, in response to starvation conditions.^12–14^

**Table 1.** Phylogeny of bacteria from the Firmicutes phylum discussed in this review

Phylum	Class	Family	Genus	Species
Firmicutes	Bacilli	Bacillaceae	*Bacillus*	*anthracis*
				*cereus*
				*circulans*
				*coagulens*
				*intestinalis*
				*lichenformis*
				*subtilis*
				*thuringiensis*
				*weihenstephanensis*
			*Geobacillus*	*stearothermophilus*
	Clostridia	Clostridiales	*Clostridioides*	*difficile*
			*Clostridium*	*bolteae*
				*botulinum*
				*butyricum*
				*disporicum*
				*freundii*
				*leptum*
				*perfringens*
				*scindens*
				*spiroforme*
				*sporogenes*
		Eubacteriaceae	*Eubacterium*	*eligens*
				*rectale*
		Lachnospiraceae	*Blautia*	not specified
			*Coprococcus*	*comes*
			*Sellimonas*	*instestinalis*
		Ruminococcaceae		
			*Ruminococcus*	*albus*
				*bromii*
	Erysipelotrichia	Erysipelotrichaceae	*Turicibacter*	*sanguinis*

The ability to survive in unfavorable environments has proven advantages and disadvantages in the fields of biotechnology and health. *Bacillus* spores have been suggested as a method for vaccine delivery^15^ or enzyme display and stabilization^16^ and are used as biodosimeters and biocontrol agents in food and agriculture^17–19^ . However, the same characteristics have led to challenges in health and disease, as the ability to form spores is linked to pathology, including persistent, chronic infection, ^20,21^ resistance to antibiotics and the development of the resistome, defined as the collection of antibiotic-resistant genes in a community.^22^

Due to the significance of spore-formers to human health and disease, it has been suggested that they be looked at as a separate grouping in microbiome studies, similar to the resistome. The term sporobiota has been suggested to cover the entirety of spore-forming bacteria in a microbial population, while the term sporobiome should be used to define a collection of genomes of spore-forming bacteria related to a particular niche.^23^

**Figure 1.**
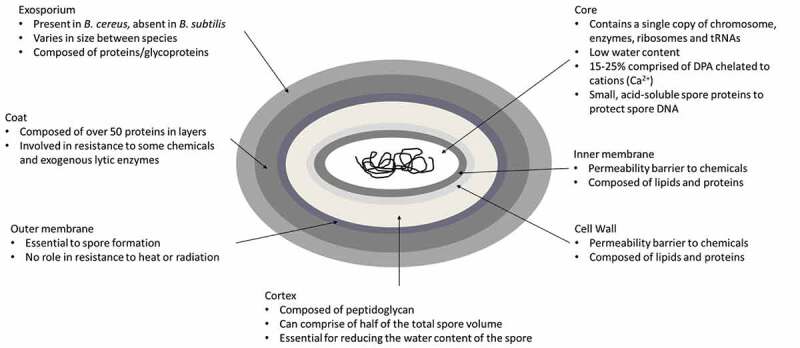
Schematic representation of an endospore of *Bacillus cereus*.^3^ The spore layers are not drawn to scale

As mentioned above, *Bacillus subtilis* is considered a model organism for endospore formation.^24,25^ Unlike the exospores and myxospores described above, endospores are formed within the mother cell which then lyses, releasing the spore.^24^ The ability to form an endospore depends on the presence of a core set of at least 60 to 100 genes which are specific to the endosporulating species of the Firmicutes phylum. Mutations in these genes can lead to a reduced or inability to sporulate.^7,26,27^ The master regulator of endosporulation is the *spo0A* gene, encoding a transcriptional regulator, which is absent in non-sporulating species and outside the Firmicutes phylum.^26,28^ The structure of the endospore is relatively conserved across species, consisting of a core compartment that contains a single copy of the genome, as well as enzymes, ribosomes and tRNAs. This is surrounded by the inner membrane and germ cell wall which are enveloped by two protective structures, namely the cortex peptidoglycan and the protein coat which are themselves separated by an outer membrane. In some species, usually those of *Bacillus cereus sensu lacto*, the protein coat is also surrounded by an exosporium (Figure 1).^29^ Fifteen to twenty-five percent of the dry weight of the spore consists of dipicolonic acid (DPA) which protects the spore DNA from external stressors. DPA is chelated to divalent cations, mostly Ca^2+.3^ A group of small, acid-soluble spore proteins (SASP) are also essential in spore resistance. These proteins are only found in the spore core, where they saturate the spore DNA, altering its structure and protecting it from heat, certain chemicals, and UV radiation.^3^

The human adult gut microbiota is dominated by the Firmicutes and Bacteroidetes phyla. The Firmicutes include spore-forming members of the Clostridiaceae, Bacilliceae and Lachnospiraceae families, as well as non-spore formers such as Lactobacillaceae and Staphylococcaceae.^30^ The infant gut is dominated by members of the Actinobacteria, Firmicutes and Bacteroidetes phyla. Maternal-infant vertical transmission is considered an important process for bacteria to reach and colonize the infant gut. Mode of feeding, gestational age and antibiotic treatment of the mother and/or infant also significantly affect the composition and development of the infant gut microbiome, ^31,32^ while C-section born infants have a significantly different microbial profile compared to their vaginally born counterparts.^32^ The purpose of this review is to investigate the sporobiota in the maternal and infant gut, as well as in important food sources for the developing infant, namely breast milk and infant formula. Due to the prevalence and influence of Firmicutes in the gut microbiome, the review will primarily concentrate on endospore-forming bacteria.

## The sporobiota of the adult gut

For a number of reasons, the abundance of spore-formers in the human gut microbiota is thought to be under-represented in many metagenomic studies. Reasons include the resistance of endospores to traditional DNA isolation techniques, the high similarity between the 16S ribosomal RNA (rRNA) and housekeeping genes of otherwise unrelated spore-formers and the fact that spore-formers tend to have larger genomes, resulting in fewer reads per gene per taxon.^23,26,33,34^

The adult gut microbiome is dominated by members of the Bacteroidetes and Firmicutes phyla but at species level is highly variable among individuals.^35,36^ The gut microbiome is heavily influenced by diet, ^37^ age, ^38,39^ obesity^40,41^ and other health disorders such as cancer and inflammatory bowel disease (IBD).^42,43^ The influence of the gut microbiota on host health and disease was reviewed comprehensively by Kho and Lal in 2018.^44^ However, the majority of studies are based on culture-independent methods such as 16S rRNA amplicon sequencing or shotgun metagenomics sequencing which, for the reasons outlined above, may underestimate the number of spore-formers present in the gut.

More recent studies have complemented culture-independent methods with “culturomics,” whereby the natural conditions and nutrients available in the gut environment are replicated *in vitro* in order to culture those microbes previously thought to be “unculturable.”^45^ The culture-dependent and independent study by Browne *et al*. was the first to use this method to specifically highlight the potential spore-formers of the adult gut microbiome. Fresh fecal samples from six healthy adults were investigated by culture-dependent and independent methods. Using a spore gene signature, the authors found that 60% of the genera present in the gut contained spore-forming bacteria and these genera represented 30% of the total intestinal microbiota. A meta-analysis of publicly available datasets revealed that this proportion remained consistent across other cohorts. In the culture-dependent approach, the fecal samples were treated with ethanol which led to the isolation of 66 distinct ethanol-resistant, possibly spore-forming species distributed across seven families. The majority of species belonged to the *Clostridium* genus but also included species from the *Ruminococcus, Blautia* and *Coprococcus* genera, previously thought to be non-spore forming.^46^ However, in a culturomics approach, the selection of the appropriate culture conditions is essential. Previous approaches using culturomics found that the Ruminococcaceae, Lachnospiraceae and Erysipelotrichaceae families, all of which have spore-forming members, could only be detected using culture-independent methods, although species belonging to the *Clostridium* and *Bacillus* genera were isolated.^47^

A recent study utilized a culture-independent approach to investigate the “resistant” fraction of the gut microbiome, particularly the spore-formers and other lysis-resistant bacteria. Fecal samples were subjected to a series of lysis treatments to select for resistant bacteria and compared to the untreated counterparts using 16S rRNA amplicon sequencing. Perhaps unsurprisingly, the resistant fraction was dominated by classes that contain spore-formers, Clostridia, Erysipelotrichia, and Bacilli. However, a particularly interesting finding was that the resistant OTUs (rOTUs) were more likely to be found in multiple individuals, as compared to the nonresistant OTUs (nOTUs) although the rOTUs were less abundant. rOTUs were also more likely to correlate with each other, with the authors suggesting that they respond coherently to environmental signals.^48^

In 2019, Forster *et al*. published the Human Gastrointestinal Bacteria Culture Collection (HBC), a collection of 737 isolates from the human gut.^49^ Of these, 496 isolates are from the Firmicutes phylum. The authors combined the genomes of these isolates with 617 publicly available, high-quality human gut-associated bacterial genomes published on the National Center for Biotechnology Information to generate the Human Gastrointestinal Microbiota Genome Collection (HGG). Functional analysis of the HGG revealed that spore-formation was over-represented among Firmicutes, demonstrating a key role for endospore-formers within this phylum in the gut. Of particular interest was that the HGG contains genomes from 173 species that were not previously isolated from the human gut, 105 of which are novel species. 85.8% of these novel species are predicted to be spore-formers, based on the genomic signature previously described by Browne *et al*. .^46,49^ This may concur with the opinion that spore-formers in the gut were previously under-represented.^23,26,33,34^

On the other hand, a recent publication by Browne *et al*. investigated the loss of sporulation in the Firmicutes of the adult gut microbiome.^50^ Genomes with a low sporulation signature score were designated as Former Spore-Formers (FSF), based on the belief that sporulation evolved just once in Firmicutes, while those with a high score were designated Spore-Formers (SF).^5,7,46^ Genomes from the Lactobacillales order were entirely FSF, while in the Lachnospiraceae (described in more detail below), just 18% were FSF. FSF genomes were associated with broader genome decay, not just in sporulation genes, indicative of host adaptation. However, while the FSF genomes were more abundant in the gut, they were less prevalent across samples, indicating that a loss of sporulation ability limits the transmission of FSF bacteria.^50^

### The clostridiaceae family

Among the spore-formers in the human gut microbiota are members of the Clostridiaceae family. This includes the genus *Clostridium*, members of which are strictly anaerobic spore-formers. Although certain species such as *Clostridium difficile* (now referred to as *Clostridioides difficile*) and *Clostridium perfringens* are known for their pathogenicity, most of the Clostridia observed in the gut have a commensal relationship with the host.^51^ In fact, *Clostridium* clusters IV and XIVa, the *Clostridium leptum* and *coccoides* groups, respectively, have been suggested to be involved in the prevention of IBD.^52^ In mouse models, *Clostridium* clusters IV and XIVa were shown to induce regulatory T-cells and suppress symptoms of dextran sodium sulfate (DSS)–mediated colitis.^53^ In a follow-up study, it was found that chloroform-resistant bacteria belonging to spore-forming *Clostridium* clusters also induced regulatory T-cells *in vitro* and in mouse models.^54^ In a study of the spore-forming contingent of the multiple sclerosis-associated microbiome, spore-forming *Clostridia* and *Bacilli* significantly reduced the severity of experimental allergic encephalomyelitis in a murine model.^55^

In one of the early shotgun metagenomics studies of the adult gut microbiome, members of the *Clostridium* genus including *Clostridium leptum* and *Clostridium scindens* were among the most abundant species in greater than 90% of individuals.^35^ Similarly, in a previous study using 16S rRNA amplicon sequencing, the spore-formers *Clostridium spriroforme* and *Clostridium bolteae* were among the 10 most frequent OTUs, present in over 50% of individuals .^56^ In a study of elderly subjects in Ireland, *C. perfringens* was observed in 7.6% of individuals, a proportion that rose to 71.4% when only individuals in long-stay residential care were analyzed. Of particular interest was the fact that the levels of bifidobacteria and lactobacilli were decreased in subjects in the *C. perfringens –* positive samples, indicative of an overall less healthy microbiome.^57^ *C. perfringens* is also overrepresented in the gut microbiome of MS patients.^55^ *C. difficile* can reside asymptomatically in the intestinal tract of humans.^58^ However, its ability to form spores is a key characteristic of its pathogenicity. *C. difficile* spreads via fecal-oral transmission and the ability to form spores allows the bacteria to survive aerobic conditions during transmission. Spore-forming also allows *C. difficile* to survive and proliferate following antibiotic treatment.

### The lachnospiraceae family

The Lachnospiraceae family is a common constituent of the gut microbiome. It consists of 24 genera, all of which are strictly anaerobic and some of which are spore-formers.^5,59^ Relative abundance of Lachnospiraceae varies depending on the stage of life, with the highest found in the adult GIT, followed by infants and with the lowest percentage in newborns.^60^ Even though the Lachnospiraceae are distributed widely throughout the environment, including other mammals, the full complement of genes required for sporulation are found only in those isolated from the human gut. The sigma-factors required to control sporulation are found in all members of the Lachnospiraceae family; however, it seems to be only those associated with the human gut which are active spore-formers.^60^ Members of the Lachnospiraceae family can be found throughout the digestive tract of humans, for example, in one study, the *Coprococcus* genus was found in both the oral cavity and stool of over 45% of the individuals tested.^61^ In a study of over 150 individuals in Michigan, OTUs belonging to the Lachnospiraceae family were part of a core microbiome, being present in over 95% of subjects. The authors suggested anaerobic Gram-positive spore-formers represent a considerable fraction of each individual’s microbiota.^62^

The Lachnospiraceae have been associated with both positive and negative health effects. In murine models, an increased abundance of Lachnospiraceae after antibiotic treatment was associated with obesity.^63^ On the other hand, Lachnospiraceae isolates formed part of a stool substitute that treated colitis caused by *C. difficile*^64^ Laffin *et al*. found that increased levels of Lachnospiraceae correlated with patients staying in remission following ileocolonic resection, a surgery used to treat Crohn’s disease. Specifically investigating the Firmicutes phylum, the authors found that individuals who remained in remission had higher numbers of anaerobic spore-formers. In contrast, those who had a recurrence of the disease had higher numbers of aerobic Firmicutes.^65^

### The bacillaceae family

Aerobic spore-formers found in the gut microbiota include members of the Bacillaceae family.^23^ Spore-forming bacilli are more commonly associated with the soil microbiome and their presence in the gut is associated with ingestion of food and water. However, despite it not being their natural habitat, *Bacillus* species are well adapted to survive the GIT and reports suggest that describing *Bacillus* as merely a “transient” member of the gut microbiome is incorrect and that they may be gut colonizers.^66,67^ A number of facultative anaerobic *Bacillus* spore-formers have been isolated from human fecal samples.^66,68,69^ In a murine model, it was shown that *Bacillus* spore-formers can undergo a complete life-cycle within the GIT, including germination, vegetative growth and re-sporulation.^68^

Similar to the Clostridia described above, *Bacillus* species have been associated with both positive and negative health effects. *Bacillus intestinalis* is a spore-former isolated from a patient with intestinal cancer.^69^ *Bacillus cereus* is associated with gut disorders such as diarrhea and irritable bowel syndrome.^70^ *B. cereus* is also a food-borne pathogen, in which the ability to form spores allows them to survive gastric transit to reach the GIT.^71^ Members of *Bacillus* spp. particularly, *B. cereus, Bacillus weihenstephanensis, Bacillus anthracis*, and *Bacillus thuringiensis* species are known to produce various toxins and are associated with food-borne intoxications.^72^ On the other hand, *Bacillus subtilis* has been shown to promote the development of gut-associated lymphoid tissues (GALT). Interestingly, the authors found that this effect was sporulation-dependent, as *spo0A* mutants were incapable of promoting GALT development. It was suggested that sporulation allows the *B. subtilis* cells to survive in the gut long enough to promote GALT development.^73^ In murine models, *B. subtilis* was found to ameliorate the effects of a DSS-induced ulcerative colitis.^74^ *Bacillus coagulens* has long been used as a probiotic with the ability to suppress the growth of pathogens, stimulate the growth of beneficial bifidobacteria and have immune-modulating effects.^75,76^ Similarly, in an M-SHIME intestinal model, a probiotic mixture of five spore-forming *Bacillus* strains was found to increase numbers of bifidobacteria and lactobacilli, as well as butyrate-producing bacteria.^77^ Indeed, the ability to form endospores is considered an advantage for *Bacillus* probiotics, as it allows them to survive gastric acidity and reach the intestine.^78^

### Potential spore formers in the adult gut

Among the other gut inhabitants belonging to the Firmicutes phylum, the genus *Ruminococcus* is generally thought to be non-sporeforming.^79^ However, in a recent study, five strains of *Ruminococcus bromii* were shown to encode most of the core sporulation genes found in *Clostridium* and *Bacillus*, and one of the strains was shown to produce spores that survive aerobic conditions.^80^ Ethanol-resistant Ruminococci have been isolated from adult fecal samples.^45^ Other *Ruminococcus* species encode sporulation genes on their genomes, even if they have not been shown to sporulate *in vitro*, for example, *Ruminococcus albus*.^5^ *Flavonifractor*, a genus also belonging to the Ruminococcaceae family can also produce ethanol-resistant spores.^46^ Using a sporulation gene signature, Abecasis *et al*. also predicted that common members of the human gut microbiota such as *Eubacterium rectale* and *Eubacterium eligens* may also be capable of endospore formation and ethanol-resistant strains have been isolated.^7,46^ Other members of the gut microbiome and members of the Firmicutes phylum are asporogenous, including the lactic acid bacteria. It has been suggested that their adaption to the nutrient-rich gut environment led to the loss of ability to form spores.^5,50^

## The sporobiota of the infant gut

### Vertical-transmission of spore-formers

Spores are specialized for host-host transmission which makes them ideal for colonization of the developing infant gut microbiota.^23^ This hypothesis was tested in a culture-independent and culture-dependent study of 40 mother-infant pairs, in which the authors specifically looked for shared spore-formers between mother and infant. Fecal samples from mothers and infants (at four different time points) were treated with ethanol and ethidium monoazide (EMA) to remove non-spore formers and their DNA. The resulting samples were analyzed by 16S rRNA amplicon sequencing. After ethanol-EMA treatment, spore-formers from the Clostridia class had a higher relative abundance in the maternal sample as compared to the infant samples at 1 week and 4 months. By 1 year, the Clostridia in the infant sample had reached a similar level to that of the maternal sample. The OTUs detected in the earliest infant samples were found to be mostly persistent in all infant samples thereafter but there was no correlation in the occurrence of these OTUs between mother and infant. In the same study, a culture-dependent approach was taken to isolate ethanol-resistant strains from eight of the mother-infant pairs. The majority of the anaerobic isolates were members of the Clostridia class with the exception being six isolates of *Turicibacter*. The aerobic isolates were identified as *B. thuringiensis* and *Bacillus circulans*. Three isolates were shared between mother and infant, namely a *Turicibacter sanguinis, Sellimonas instestinalis* and a *Clostridium disporicum*. The authors concluded from this study that there is a low level of sharing of spore-formers between mother and infant by vertical transmission and spore-formers are more likely to have an environmental source.^81^

A similar hypothesis was made following a study by Nayfach *et al*.^82^ They found that shared strains between mother and infant in the days immediately after birth belonged to the *Bacteroides vulgatus, Parabacteroides distasonis, Bifidobacterium adolescentis* and *Escherichia coli* species, which are non-spore forming. In contrast, species with low rates of vertical transmission tended to have a higher sporulation score, based on the presence of sporulation genes on their genomes. This supports the notion that sporulating bacteria tend to be acquired from environmental sources rather than from vertical transmission.^83^

A number of other studies have investigated vertical transmission of strains without specifically examining sporulation ability. In a large study of mother-infant pairs using 16S rRNA amplicon sequencing, it was observed that OTUs belonging to the Clostridia and Erysipelotrichia classes persistently colonized over 50% of the mothers in the study. However, when the authors investigated the shared OTUs between mother-infant pairs, the number of Clostridial OTUs dropped considerably and was instead enriched with non-spore-forming Bacteroidia. It was suggested that the Clostridia are more likely to be “late colonizers,” as opposed to those acquired through vertical transmission at birth.^83^ In a study of vertical transmission between mothers and infants, 62 strains were found to have strong evidence of vertical transmission but only *R. bromii* were potential spore-formers.^84^ In a similar study, strains of *Coprococcus comes* and *R. bromii* were identified in mother-infant pairs.^85^ As mentioned above, members of the *R. bromii* species can encode sporulation genes on their genomes and certain strains can form spores.^46,80^ Meanwhile, *Cop. comes* encodes the *spo0A* gene as well as germination receptors and it has been speculated that members of this species may sporulate under unusual conditions, although it has not been proven *in vitro*.^86^ On the other hand, a separate study using the same method of identifying single nucleotide variants found that only strains from the non-spore-forming classes, Actinobacteria and Bacteroidia, were shared between mothers and infants in the first days of life. Maternal strains belonging to the Clostridia class were not shared with the infant. The expansion of Clostridia, later on, was attributed to environmental sources rather than maternal.^87^ Finally, in a Finnish cohort, just five bacterial species were common between mother and infant at an abundance higher than 5%, three *Bacteroides* species, two *Bifidobacterium* species and *E. coli*, none of which are spore-formers.^88^

### Development of the sporobiota over time

Aside from vertical transmission, a number of other factors can affect the colonization and development of the infant gut. One such factor is the age of the infant, which has a profound effect on the composition of the microbiome and indeed the prevalence of potential spore-formers. The Lachnospiraceae tend to colonize the gut microbiome from approximately 1 year after birth but prior to that, the microbiome is dominated by non-spore-forming Bacteroidaceae and Bifidobacteriaceae. Since the Lachnospiraceae family contains spore-forming genera, it has been suggested that the increase in sporulating ability over time is to allow increased dispersal among hosts or persistence within the host during stressful conditions.^89^ A number of other studies also found an increasing abundance of Lachnospiraceae with age.^90–92^ It is possible that the increasing abundance of Lachnospiraceae with time is indicative of the transition toward the adult-like microbiome, particularly since this family is negatively correlated with exclusive breastfeeding.^91^ In a systematic review, Lachnospiraceae were shown to be a dominant family in older children of 8 years and older.^93^ This age-related colonization by the Lachnospiraceae family would suggest that it is not commonly passed to the infant at birth via vertical transmission.

The research is divided as to when the *Clostridium* genus colonizes the infant gut. As mentioned above, certain studies describe the Clostridia class as late colonizers.^83,87^ However, other studies have found the *Clostridium* genus as early as day one or week one in the infant gut, although significantly higher in C-section born infants (see below).^32,94,95^ *C. difficile*-colonized infants have significantly altered microbiota profiles as compared to non-colonized infants, regardless of the mode of feeding, age and gestational age.^96^

In a recent study, the Clostridiaceae family was dominant among endospore-producing families in the infant gut microbiome. However, as above, the Lachnospiraceae, as well as the Peptostreptococcaceae and Erysipelotrichaceae increased with time, being significantly higher at 360 days compared to 90 and 180 days. At 90 days, endospore-forming *Clostridium senso stricto* was the highest of the butyrate-producing bacteria present in the infant gut microbiome.^97^ Low levels of butyrate, a short-chain fatty acid and product of glycolysis have been linked to allergic diseases later in life.^98,99^

### Sporobiota, mode of delivery and antibiotic treatment

Delivery mode is well known to have a significant effect on the bacterial composition of the infant gut.^31,32^ In terms of spore-formers, the *Clostridium* genus is found at a significantly higher relative abundance in C-section-delivered infants as compared to their vaginally born counterparts in the early weeks of life.^32,100^ *C. difficile, Clostridium g4* and *C. perfringens* are increased in C-section born infants.^49,101,102^ The KOALA study in the Netherlands identified C-section delivery as a risk factor for the development of atopic disease, specifically due to the overrepresentation of *C. difficile*. Indeed, *C. difficile* is consistently over-represented in allergic children.^103,104^

An over-representation of the *Clostridium* genus in C-section born infants also has a significant and interesting effect on the other typical members of the infant gut microbiota. According to multiple studies, the *Bacteroides* genus is most affected by the mode of delivery. The relative abundance of this genus is significantly lower in C-section-born infants in the first week of life compared to their vaginally born counterparts, a discrepancy that continues into infancy (6 to 8 months).^31,32^ Nagpal *et al*. discovered a negative correlation between *C. perfringens* and *Bacteroides fragilis*. Infants colonized by *C. perfringens* early in life (from birth to 6 months) tend to have lower numbers of *B. fragilis*, as well as bifidobacteria.^102^

Intrapartum antibiotic prophylaxis (IAP) to prevent wound infection is recommended for mothers undergoing C-section or to prevent the transmission of Group B *Streptococcus* during vaginal delivery.^105,106^ Maternal IAP is associated with an over-representation of the *Clostridium* genus in infants, irrespective of birth mode.^107^ Likewise, antibiotic treatment of vaginally born infants in the first days of life also leads to an increase in *Clostridium*.^107^ However, antibiotic treatment in infants has been linked with a decrease in other spore-forming taxa, perhaps surprisingly given that spore-formers are typically resilient to antibiotics. Guittar et al. showed that infants exposed to repeated antibiotic treatment had fewer gut taxa capable of sporulation such as Lachnospiraceae.^89^ Bokulich et al. showed that antibiotic-treated infants have a delayed microbiome maturation, specifically due to the depletion of specific OTUs from the Lachnospiraceae and Erysipelotrichaceae families.^108^

### Sporobiota and gestational age

Preterm infants face a number of challenges in terms of microbiome development, including rapid vaginal or C-section deliveries reducing exposure to the maternal microbiota, repeated antibiotic treatment, prolonged hospitalization and supplementary formula feeding.^109^ Such challenges significantly affect the composition and development of the gut microbiome.^32^ The *Clostridium* genus comprises 10% of the premature infant gut, as compared to 5% in term infants during the first 6 weeks of life. The Lachnospiraceae were not affected by gestational age, representing 8% of the population in both term and preterm infants.^110^ At a species level, *C. difficile, C. perfringens* and *Clostridium freundii* were identified at a relative abundance greater than 1% in 144 preterm infants.^111^ *Clostridium senso stricto* were found to be seven times more abundant in preterm infants suffering from early-onset necrotizing enterocolitis (NEC).^112^ Similarly, using a combination of 16S rRNA amplicon sequencing and culture-based approaches, *Clostridium butyricum* was found to be significantly associated with NEC in 15 preterm infants.^113^

### Sporobiota and mode of feeding

Mode of feeding as well as the introduction of solid foods may have an impact on the abundance of potential spore-formers in the infant gut. *C. difficile* is typically higher in formula-fed compared to breast-fed infants.^114^ In a study of 98 mother-infant pairs, formula-fed infants had elevated levels of *C. difficile* in comparison to breast-fed infants at 4 months. The cessation of breastfeeding at 12 months also led to an increase in levels of *Clostridium*.^115^ The *Clostridium coccoides* group of spore-formers is also higher in formula-fed infants and post-weaning, even in infants that were exclusively breastfed prior to the introduction of solid foods.^116^ This correlated with a large Danish study that found that cessation of exclusive breastfeeding led to a microbiota dominated by *Clostridium* species.^117^ As mentioned previously, multiple studies have found that the Lachnospiraceae are negatively associated with exclusive breastfeeding and positively associated with the post-weaning period.^100,118,119^

### Sporobiota of breast milk

As described above, spore-forming bacteria tend to be more abundant in formula-fed infants as compared to their breastfed counterparts. Reports on the breast milk microbiome vary widely but data indicate that spore-formers are rarely present.^120^ Instead, it is dominated by members of the Proteobacteria or Firmicutes phyla, namely Staphylococcaceae and Streptococcaceae. *Lactobacillus* and *Bifidobacteria* have also been identified in breast milk but results have varied between studies.^120–123^ Two studies have found members of the Lachnospiraceae in breast milk but it is not known if this included spore-forming genera.^124,125^ On rare occasions, members of the *Bacillus* genus are identified but at a low relative abundance, although the authors noted that their presence was positively correlated with higher protein content.^126^ Surprisingly given their status as strict anaerobes, the *Clostridium* genus has on occasion been identified in breast milk but at a low relative abundance (less than 1%) and only by culture-independent methods.^127^

Although not typically found in breast milk microbiome studies, *B. cereus* spores represent a significant problem in pooled breast milk samples. In neonatal intensive care, premature infants can be fed donor human milk to supplement the mother’s breast milk. Pooled breast milk has been implicated in serious or fatal *B. cereus* infections in premature infants, although other case reports have suggested that the source is more likely the hospital environment.^128–130^ Using culture-dependent methods, *B. cereus* was identified in 9.2% of 152 raw donor milk samples.^131^ Donor milk is pasteurized for 30 min at 62.5°C, also known as the Holder method.^132,133^ However, multiple studies have shown that this method is not always effective in killing *Bacillus* spores. In a study of 303 pooled milk samples, 5% were positive for *Bacillus* after pasteurization.^134^ A later study had similar results, of 190 milk cultures tested, 5.8% tested positive for *Bacillus* post-pasteurization.^133^ No other bacteria were detected post-pasteurization, indicating that it is the spore-forming ability of the *Bacillus* isolates that affords them the ability to survive pasteurization. Another study suggested that the numbers of *B. cereus* post-pasteurization may actually be under-represented, as the spores need to germinate in order for them to be identified by typical culture-based methods. The authors incubated the post-pasteurized samples for 18 h at 37°C to encourage germination and found that the number of samples positive for *B. cereus* increased from 3.3% to 10.7%.^129^ New methods to deactivate *Bacillus* spores are being investigated, such as a high-hydrostatic pressure process which resulted in a six log reduction of *B. cereus* spores.^132^

### Sporobiota of infant formula

Aerobic spore-formers also have a significant impact on the safety and quality of powdered infant formula (PIF), due to their ability to survive extremes in heat, dryness and disinfectants. PIF is not a sterile product but is required to reach high standards of microbiological quality. *Cronobacter* species and *Salmonella enterica* are listed by the World Health Organization (WHO) as the pathogens of most concern in PIF, while the spore-formers *B. cereus, C. difficile, C. perfringens, C. botulinum* are also listed among the primary microorganisms associated with PIF contamination.^135^ Bovine milk forms the base of almost all PIF and is supplemented with protein, lipids and carbohydrates. PIF can be produced in three different processes, wet-mix, dry-mix or combined. In the wet-mix process, the components are blended and the formulation is pasteurized followed by spray-drying. In the dry-mix approach, the components are individually pasteurized and dried. The individual dry components are then mixed and dispensed into the final packaging. The combined approach is a combination of the wet-mix and dry-mix processes.^136^ Given the high degree of aeration involved in dairy powder processing, *Bacillus* species tend to be more prevalent than anaerobic *Clostridium*.^137^ Similar to pooled breast milk, spores can germinate post-sterilization, resulting in high numbers of vegetative cells in the end product.^138^

As in microbiome studies, identifying and quantifying spore-formers in PIF presents a number of challenges. In Europe, testing for *B. cereus* involves plating on Mannitol Egg Yolk Polymyxin (MYP) agars and the hemolysis test. However, this has limitations in selectivity, accurate identification and is labor- and time-consuming. More recently developed protein and DNA-based tests have been suggested to provide more robust data.^137^

China is one of the world’s largest producers of PIF and has stringent criteria for aerobic plate counts in infant formula, with an upper limit of 10^3^ cfu/g.^139^ A study of an infant formula production facility in China identified 84 distinct *B. cereus* isolates across the whole facility. The vast majority (80 isolates) were discovered in the processing environment as opposed to the raw materials.^140^ Similarly, in a study of airborne microorganisms in a PIF production facility, *Bacillus* was among the dominant genera, with *B. lichenformis* being the second most dominant species behind *Staphylococcus epidermis*. These species were found at multiple locations across the production facility.^141^

Aerobic spore-formers are also found in PIF products off the shelf. In a Swiss study of nine different PIF brands, 78% of products had *Bacillus* spores.^142^ In a wider study of 25 milk powder products from across China, including 12 infant formula products, 9 were over the aforementioned limit for thermophilic spore-formers and 10 were above the limit for mesophilic spore-formers. *B. lichenformis* was the dominant species, identified in 23 of the 25 samples and represented 43% of all the isolates identified.^139^ Anaerobic spore-formers have also been isolated from shelf products, including *C. botulinum, C. perfringens* and *Clostridium sporogenes*.^143^

Many of the spore-formers identified in PIF are nonpathogenic and their presence is more indicative of poor hygiene processes.^136^ However, the potential for the serious disease cannot be underestimated. Infant botulism has been linked to infant formula contaminated with *C. botulinum* spores.^144^ A relatively unexplored topic is the potential for spore-forming *Bacillus* to produce nitrite during PIF processing. Nitrate is a natural contaminant in milk powder but in the form of nitrite can cause methemoglobinemia which can be fatal in infants. A study by Cho and Rhee found that a number of *Bacillus* and *Geobacillus* spore-formers isolated from PIF processing plants were capable of converting nitrate to nitrite.^145^

## Conclusions and future perspectives

Spore-forming bacteria are ubiquitous throughout nature, with their prevalence perhaps even under-estimated in many environments. However, it is only in recent years that human microbiome studies have specifically focused on the spore-forming members of this bacterial community. It is now known that the sporobiota constitute a significant part of the human microbiome, in terms of population and influence. These studies have also brought to attention the potential spore-forming ability of common gut taxa such as *Ruminococcus*, previously thought to be non-spore-forming. The prevalence of sporulation gene signatures and the ability to produce spores among these taxa certainly merit further investigation.

In terms of the adult gut microbiome, as much as 50% of the bacterial community have spore-forming potential and are more likely to be shared among individuals. This is particularly relevant to human health, given the prevalence of antibiotic resistance genes in spore-forming bacteria. The ability to form spores is also a key trait in the spread and recurrence of *Clostridium* infections. It would be interesting to investigate the overlap of the resistome and sporobiota of the human gut in tandem, to see how these two groups may overlap.

In the infant gut, studies would indicate that spore-formers are “late colonizers,” increasing in abundance as the infant microbiome moves closer to that of the adult. However, as numerous studies have shown, the infant gut microbiome is heavily influenced by factors such as mode of delivery, mode of feeding and infant age, factors that also affect the sporobiota. Extrinsic factors such as spore-formers found in pooled breast milk and infant formula may also aid transmission and colonization of the sporobiota in infants. The effects of spore-formers on infant health cannot be over-estimated, whether it be the more immediate effects of infection or long-term affects such as allergy and will likely be the subject of further investigation.

The developments in culture-independent methods such as the decreasing costs of shotgun metagenomics sequencing, improvements in machine learning, functional analyses, as well as the rebirth of culture-dependent methods through culturomics, mean that no bacterial group should be considered out of reach for a thorough investigation. Given the potential for spore-forming bacteria in health and disease, we may be just skimming the surface of this crucial niche.
